# Body Roundness Index Outperforms Body Mass Index in Predicting Obstructive Sleep Apnea Severity Among Chinese Adults

**DOI:** 10.3390/jcm14248764

**Published:** 2025-12-11

**Authors:** Ningchang Tang, Yuenan Ni, Fengming Luo

**Affiliations:** 1 Department of Respiratory and Critical Care Medicine, West China School of Medicine and West China Hospital, Sichuan University, Chengdu 610041, China; keroppi_tt@163.com (N.T.); vivian940305@foxmail.com (Y.N.); 2 Department of Respiratory Care, West China Hospital, Sichuan University, Chengdu 610041, China

**Keywords:** obstructive sleep apnea, body roundness index, stop-bang questionnaire, visceral adiposity, screening tool, cardiometabolic risk, Chinese population

## Abstract

**Background**: Obesity is a key factor in obstructive sleep apnea (OSA), though Body Mass Index (BMI) may not fully capture this risk as it ignores visceral fat distribution. The Body Roundness Index (BRI), which uses waist circumference and height to better reflect visceral adiposity, has not been widely validated for OSA screening. This study assesses a BRI-based model for predicting severe OSA. **Methods**: Patients undergoing polysomnography (PSG) were retrospectively enrolled from January 2022 to March 2025 and prospectively enrolled from March 2025 to June 2025. Least absolute shrinkage and selection operator (LASSO) regression was used to identify optimal predictors of severe OSA. A predictive model for severe OSA (Apnea–Hypopnea Index [AHI] ≥ 30 events/h) was developed using BRI and other relevant factors. Subgroup analyses were performed by sex and age. **Results**: A total of 7579 patients were included in the final analysis, of whom 3864 (51%) were diagnosed with severe OSA. Univariable logistic regression revealed that each unit increase in BRI was associated with a significantly elevated risk of severe OSA (OR 2.01, 95% CI 1.93–2.10; *p* < 0.001). The predictive severe OSA model incorporating BRI yielded higher area under the receiver operating characteristic curve (AUC) values (Training: 0.762 vs. 0.747; Test: 0.820 vs. 0.803; DeLong test *p* < 0.05). Subgroup analyses by sex and age revealed higher AUCs across all groups, with the most pronounced improvements in sensitivity observed in women (84.3% vs. 73.0%) and individuals aged ≤ 50 years (75.6% vs. 60.2%). **Conclusions**: BRI is more strongly correlated with severe OSA than BMI and may enhance screening efficacy in Chinese adults.

## 1. Introduction

Obstructive sleep apnea (OSA) is a prevalent sleep-related breathing disorder characterized by recurrent upper airway collapse during sleep, resulting in intermittent hypoxia, microarousals, and sleep fragmentation. Globally, an estimated 936 million adults are affected by OSA, with approximately 176 million cases in China, reflecting a prevalence of 23.6%, of which 37.5% are moderate-to-severe [1]. OSA is associated with significant health consequences, including an increased risk of hypertension, diabetes, cardiovascular disease, and stroke [2,3,4,5]. The rising incidence of OSA in China, driven by increasing obesity rates and population aging, poses substantial public health and economic challenges.

Obesity is a critical modifiable risk factor for OSA, as excess adiposity, particularly visceral fat, contributes to airway obstruction by increasing soft tissue mass around the upper airway and reducing lung volume [6]. In China, the growing prevalence of obesity parallels the rising incidence of OSA, amplifying its public health burden. However, the traditional body mass index (BMI) has significant limitations in assessing OSA risk. The Lancet Diabetes & Endocrinology recently redefined obesity (2025) as a “chronic disease state of excess adiposity, with or without abnormalities in adipose distribution or function,” emphasizing that BMI fails to differentiate visceral fat from muscle mass or account for fat distribution [7]. This is particularly relevant for Chinese populations, who, due to unique craniofacial anatomy (e.g., shorter mandible and maxilla, larger facial angles) [8] and a higher propensity for visceral adiposity, exhibit elevated OSA risk even at non-obese BMI levels. Studies indicate that 25–50% of Chinese patients with severe OSA (Apnea–Hypopnea Index [AHI] ≥ 30 events/h) have a BMI below the obesity threshold, highlighting BMI’s inadequate sensitivity for OSA risk stratification in this population [9,10].

The Body Roundness Index (BRI), calculated from waist circumference and height using an elliptical geometric model, offers a more precise measure of visceral fat volume and trunk adiposity distribution [11]. The BRI has shown superior predictive efficacy compared to traditional anthropometric indices for conditions such as cardiovascular disease, osteoarthritis, and colorectal cancer [12,13,14]. Despite its promise, the application of BRI in predicting OSA remains underexplored. Limited studies, primarily using National Health and Nutrition Examination Survey (NHANES) data, have investigated BRI-OSA associations, but methodological heterogeneity and underrepresentation of Asian populations prevent definitive conclusions about its predictive value in Chinese individuals [15,16].

This study utilizes a retrospective and prospective cohort design, enrolling 7579 Chinese adults from Southwest China who underwent polysomnography (PSG). We aim to validate whether the BRI outperforms BMI in screening and stratifying severe OSA, establishing BRI’s optimal threshold to enhance OSA risk identification.

## 2. Methods

### 2.1. Study Population and Design

**Figure 1:**
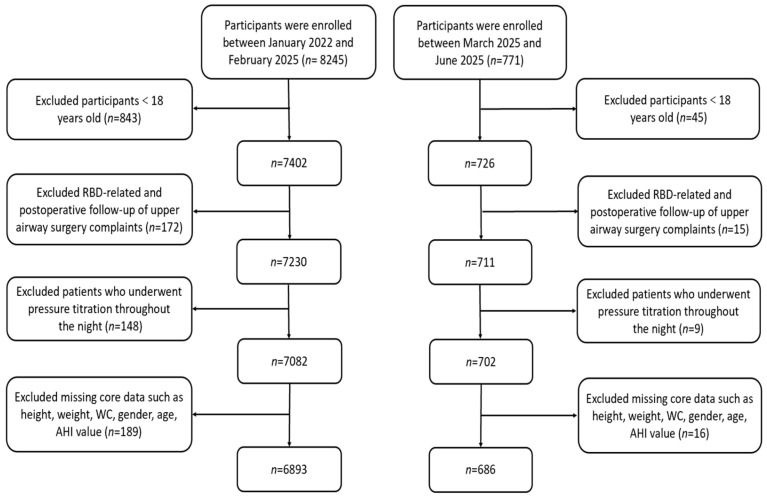
A flow diagram of eligible participant selection. Abbreviations: RBD, Rapid-eye-movement sleep Behavior Disorder; WC, Waist Circumference; AHI, Apnea–Hypopnea Index.

Consecutive patients undergoing diagnostic PSG at the Sleep Medicine Center between January 2022 and June 2025 were screened (*n* = 9016). Participants with missing data for any key baseline parameters were excluded from the analysis. No imputation was performed. After applying exclusion criteria, 7579 eligible participants were enrolled (Figure 1). Exclusion criteria were: (1) age < 18 years; (2) post-upper airway surgery follow-up; (3) patients undergoing continuous positive airway pressure (CPAP) titration. The study protocol was approved by the Hospital Ethics Committee.

### 2.2. Anthropometric Measurements and Questionnaires

Standardized anthropometric measurements included height, weight, waist circumference (WC), and neck circumference (NC). Weight was measured to the nearest 0.1 kg using a calibrated scale, with participants standing barefoot in light clothing. Height was measured to the nearest 0.1 cm using a stadiometer. WC was measured at the midpoint between the iliac crest and the costal margin at end-expiration. NC was measured at the level of the cricothyroid membrane. BMI and BRI were calculated using standard formulas:


$$BMI=\frac{weight(kg)}{{height(m)}^{2}}$$

$$BRI=364.2-365.5\times \sqrt{1-{\left(\frac{WC\left(cm\right)/\left(2\pi \right)}{0.5\times height\left(cm\right)}\right)}^{2}}$$

Daytime sleepiness and stop-breathing events were assessed using the corresponding “tiredness” and “observed apnea” items from the STOP-Bang questionnaire. Participants reporting frequent unintended dozing or excessive sleepiness during daytime activities were coded as “Yes” for daytime sleepiness, and those (or their bed partners) reporting regular breathing pauses or gasping/choking episodes during sleep were coded as “Yes” for stop-breathing [17].

### 2.3. Polysomnography

Overnight PSG was performed using Alice 6 LDxN polysomnographs (Philips Respironics, Murrysville, PA, USA) following American Academy of Sleep Medicine (AASM) version 3.0 guidelines [Scoring Manual V3.0]. Recorded parameters included: electroencephalography (EEG; 10–20 system), electrooculography (EOG), chin electromyography (EMG), electrocardiography (ECG), nasal airflow (using both nasal pressure transducer and thermistor), thoracic and abdominal respiratory effort (inductive plethysmography), pulse oximetry (SpO_2_), and body position. Apnea was defined as a ≥90% reduction in airflow for ≥10 s. Hypopnea was defined as a ≥30% reduction in airflow associated with either a ≥3% oxygen desaturation or an EEG arousal. The AHI was calculated as the total number of apneas and hypopneas per hour of sleep. Participants were categorized as: Non-OSA (AHI < 5 events/h), Mild OSA (AHI 5–14.9 events/h), Moderate OSA (AHI 15–29.9 events/h), and Severe OSA (AHI ≥ 30 events/h). All studies were independently scored by two experienced technicians. Inter-rater reliability was excellent (Kappa coefficient ≥ 0.85).

### 2.4. Statistical Analysis

Statistical analyses were performed using R software (version 4.4.1). Continuous variables were expressed as mean ± standard deviation for normal distributed data or median (interquartile range) for non-normally distributed data. The Shapiro–Wilk test was used to assess normality. Group comparisons for continuous variables were performed using the Wilcoxon rank-sum test or Kruskal–Wallis test, as appropriate. Categorical variables are presented as frequency (percentage) and were compared using Pearson’s chi-square test or Fisher’s exact test. Data collected between January 2022 and February 2025 were randomly split into training and internal validation sets. An independent test set was formed from prospectively collected data from March 2025 to June 2025. We performed baseline characteristic comparisons between the retrospective cohort and the prospective cohort.

Least absolute shrinkage and selection operator (LASSO) regression was applied with 10-fold cross-validation to select and compare variables associated with severe OSA. The penalization parameter λ was determined using the 1-SE rule. The initial predictor set included components from the STOP-Bang questionnaire (such as snoring, daytime sleepiness, hypertension, BMI, etc.) along with the BRI. Subsequently, a predictive model for severe OSA (AHI ≥ 30 events/h) was constructed by integrating BRI with other significant predictors identified, to construct the final predictive score.

To evaluate diagnostic performance, receiver operating characteristic (ROC) curves were generated for both the traditional STOP-Bang score and the BRI-optimized model in the training, validation, and test sets. Area under the ROC curve (AUC) values and 95% confidence intervals (CI) were estimated using bootstrap resampling. The DeLong test was used to compare the AUCs between models. Sensitivity, specificity, positive predictive value (PPV), and negative predictive value (NPV), with 95% CIs, were calculated at the prespecified cutoff points. The optimal cutoff for the BRI-based model was determined by maximizing the Youden index (J = sensitivity + specificity − 1), yielding a value of 3.97; to facilitate clinical use and account for measurement variability, this value was rounded to the nearest integer (BRI ≥ 4). Applying BRI ≥ 4 did not materially change the AUC or the overall sensitivity–specificity profile compared with the unrounded threshold. Subgroup analyses by sex (male/female) and age (≤50 years, >50 years) used stratified ROC analyses. All statistical tests were two-sided with a significance threshold of α < 0.05.

## 3. Results

The study included 7579 participants (71.3% male; mean age 43.9 ± 13.1 years). Baseline characteristics differed significantly between the severe OSA group (*n* = 3864, AHI ≥ 30) and the non-severe group (*n* = 3715) (Table 1). The severe OSA group had a higher proportion of males (84.4% vs. 57.7%), hypertension (29.8% vs. 13.6%), and diabetes (6.8% vs. 3.7%) (all *p* < 0.001). BMI (27.1 ± 3.9 vs. 23.9 ± 3.6 kg/m^2^), WC (96.6 ± 10.2 vs. 86.7 ± 10.8 cm), NC (39.7 ± 3.6 vs. 36.4 ± 3.8 cm), and BRI (4.9 ± 1.3 vs. 3.9 ± 1.2) were significantly higher in the severe OSA group (all *p* < 0.001). Participants classified as high-risk by BRI (BRI ≥ 4, *n* = 4559) had a significantly higher median AHI (46.2 ± 28.9 vs. 22.9 ± 21.1) and greater cardiometabolic comorbidity burden compared to the low-risk group (BRI < 4, *n* = 3020) (Table 2).

The LASSO analysis revealed that, after adjusting for other variables, BRI demonstrated superior predictive power for severe OSA compared to BMI, with its standardized coefficient approximately twice that of BMI (BRI β ≈ 0.161 vs. BMI β ≈ 0.081). Furthermore, the LASSO analysis (λ = 0.20951, using the 1SE criterion) identified the BRI-based predictive model score—optimized via the Youden index—as the strongest predictor of severe OSA (β = 0.32, *p* < 0.001), while the coefficients of all other predictive models were shrunk to zero.

The *p*-values for baseline comparison between the retrospective and prospective cohorts were mostly <0.05, suggesting significant differences. However, the prospective cohort was used as an independent test cohort to assess the external applicability of the model. Furthermore, Diagnostic performance was nearly identical at the original threshold of 3.97 and the rounded value of 4 (ΔAUC < 0.001; all DeLong *p* > 0.05), supporting the use of a clinically simplified cutoff without loss of discrimination.

**Figure 2:**
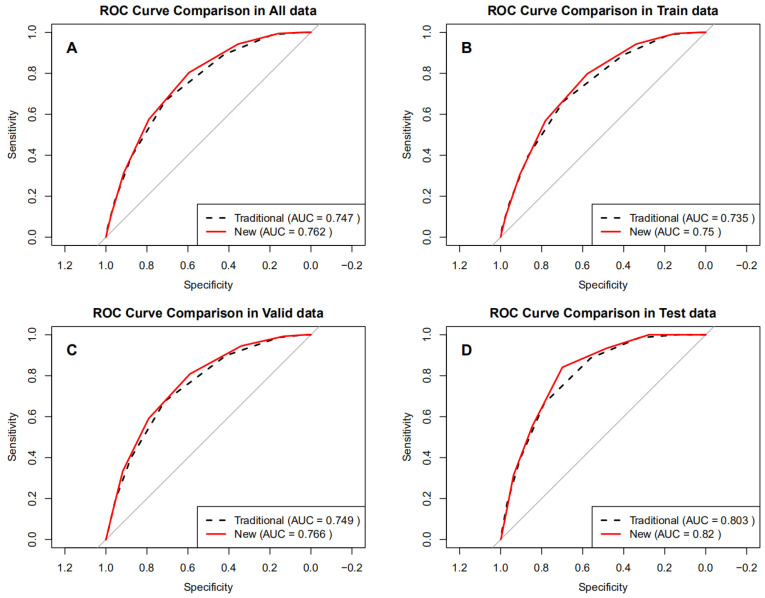
ROC curves of the optimized BRI versus the traditional STOP-Bang score in different datasets. (**A**) All data, (**B**) Train data, (**C**) Valid data, (**D**) Test data.

ROC analysis (Figure 2A–D) showed that the BRI-optimized STOP-Bang score achieved an AUC of 0.762 (95% CI 0.751–0.772), higher than the traditional score (0.747, 95% CI 0.736–0.757). DeLong’s test confirmed a statistically significant improvement (ΔAUC = 0.015, 95% CI 0.011–0.019; *p* < 0.001). This pattern was consistent across the training and validation sets (ΔAUC range 0.015–0.017, all *p* < 0.001). In the independent test set, the BRI-optimized model also showed superior discrimination (0.820 vs. 0.803; ΔAUC = 0.017, 95% CI 0.006–0.027; *p* = 0.003) (Table 3).

**Figure 3:**
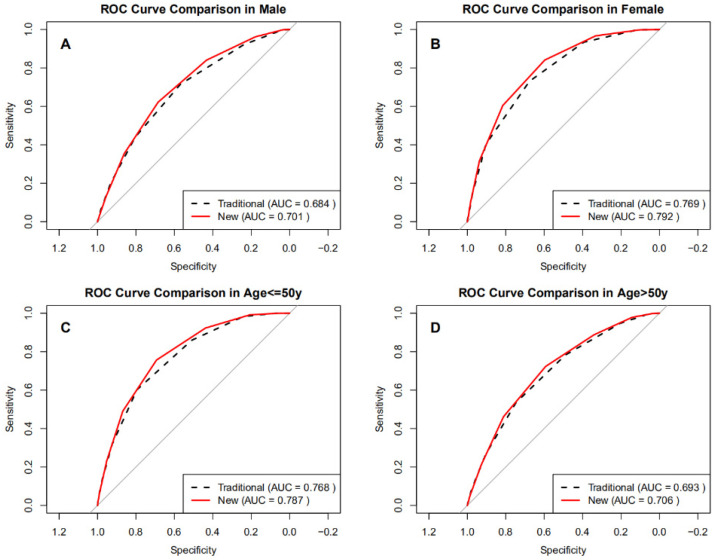
ROC curves of the optimized BRI versus the traditional STOP-Bang score in subgroups. (**A**) Male, (**B**) Female, (**C**) Age ≤ 50 years, (**D**) Age > 50 years.

Subgroup analyses assessed diagnostic performance by sex and age (Figure 3A–D). The BRI-optimized STOP-Bang model achieved higher AUCs than the traditional STOP-Bang score across all subgroups (Table 4), ranging from 0.701 to 0.792 compared to 0.684 to 0.769 for the traditional method. The BRI-optimized score produced higher AUCs than the traditional STOP-Bang. Improvements were modest but statistically significant, with ΔAUC values of 0.017 in men, 0.023 in women, 0.019 in adults ≤ 50 years, and 0.013 in those > 50 years (all DeLong *p* < 0.001). Sensitivity gains were most pronounced in women (84.3% vs. 73.0%) and younger adults (75.6% vs. 60.2%), while specificity notably improved in men (68.5% vs. 56.8%). These findings demonstrate that integrating BRI into the STOP-Bang score offers consistent, statistically robust improvements in identifying severe OSA, particularly in groups traditionally underdetected using BMI-based screening.

## 4. Discussion

Using a large clinical sample from Southwest China and PSG as the diagnostic gold standard, our findings suggest that the BRI-based predictive model provides better discrimination for identifying severe OSA compared to previous methods. The BRI-optimized model showed modest but consistent improvements in AUC across training, validation, and prospective test sets, and was particularly effective in enhancing sensitivity among women and younger adults. By employing a straightforward clinical threshold, the BRI-based tool maintains ease of use while enhancing screening efficiency, providing a practical and cost-effective solution for severe OSA detection in the Chinese population, suggesting that BRI may be a useful anthropometric indicator for refining OSA risk stratification in Chinese adults.

As a novel adiposity index, BRI combines waist circumference and height to quantify trunk fat distribution, overcoming the limitations of BMI in assessing visceral adiposity [18]. Considering the unique body composition and cardiometabolic risk factors of East Asians [19], this study modified the BMI threshold in the traditional STOP-Bang from 35 kg/m^2^ to 30 kg/m^2^ for Chinese adults and compared it against the BRI-optimized version. LASSO regression confirmed the BRI-based predicting model as the strongest predictor, outperforming the BMI-based model. Prior studies by Pan et al. [15] and Zhang et al. [16] using NHANES data found a strong positive association between BRI and OSA risk (optimal cutoff ≈ 4.3, with each unit increase raising risk by 167%), supporting its potential utility. Our cutoff (3.97, rounded to 4) is consistent with Wang et al. [9], who showed that the sensitivity of the STOP-Bang was limited in Chinese patients, especially those with lower BMI, and that adjusting BMI and NC thresholds improved performance. Similarly, Ma et al. [20] proposed adding morning dry mouth and adjusting BMI > 28 kg/m^2^ to enhance specificity, while Sangkum et al. [10] in Thai patients improved perioperative screening by modifying BMI and NC thresholds, indirectly supporting the feasibility of BRI optimization. These align with findings by Chen et al. [19] on early-life BMI and cardiovascular outcomes, further underscoring the localized applicability of BRI in East Asia.

Our findings extend previous work on the relationship between BRI and OSA. Recent population-based studies have reported that higher BRI is associated with an increased risk of questionnaire-defined OSA or OSA-related symptoms in adults, and that BRI may perform at least as well as, and in some subgroups better than, BMI for identifying individuals at high risk of OSA [21,22]. However, these studies relied on symptom-based or questionnaire definitions of OSA and did not assess BRI within multivariable screening tools or against PSG-confirmed OSA severity. In contrast, our study used overnight PSG as the gold standard in a large cohort of 7579 adults and directly compared a BRI-optimized STOP-Bang model with the traditional BMI-based STOP-Bang across development and validation datasets. Taken together, these results support the notion that BRI, as a surrogate marker of visceral adiposity, is more strongly associated with severe OSA risk than BMI in this population and can improve the performance of existing screening tools when integrated in a simple and clinically interpretable way.

Given that BRI is closely related to visceral adiposity, which is especially relevant for Asian populations, this model may have utility not only in Chinese adults but also in other Asian populations with similar body shapes. Further studies are needed to validate the model’s application in these groups.

Current OSA screening relies on traditional tools such as the STOP-Bang questionnaire, which incorporates BMI alongside other risk factors like snoring, tiredness, and hypertension. However, the STOP-Bang questionnaire’s reliance on BMI limits its effectiveness in Chinese populations, where visceral fat accumulation and craniofacial predispositions drive OSA risk independently of BMI. This results in a high rate of missed diagnoses, particularly for individuals with normal or mildly elevated BMI [6,23]. To address these limitations, alternative anthropometric measures are needed for more accurate OSA risk assessment.

The traditional STOP-Bang has been widely adopted in outpatient, community, and perioperative settings due to its simplicity, low cost, and high sensitivity [17,24]. However, its reliance on BMI > 35 kg/m^2^ contributes to underdiagnosis in Chinese populations. Our BRI-based predicting model (threshold 4) significantly improves sensitivity for severe OSA while preserving simplicity, making it highly suitable for resource-limited community screening. In a populous country like China, enhancing screening efficiency could markedly improve early identification and treatment of OSA, ultimately reducing the burden of cardiometabolic diseases [19,25,26]. Additional studies have also confirmed that BRI outperforms BMI and waist-to-hip ratio (WHR) in predicting metabolic syndrome, cardiovascular risk, and hypertension [27,28,29], providing strong theoretical support for its application in OSA.

Subgroup analysis revealed distinct advantages of the BRI-optimized tool across sex and age. Sensitivity in women improved from 73.0% to 84.1%, suggesting that BRI better captures visceral adiposity distribution in women, overcoming the limitations of BMI-based screening. Female OSA patients often present with atypical symptoms (e.g., absence of loud snoring) and lower BMI, leading to underdiagnosis by the traditional STOP-Bang [30]. Mou et al. [31] emphasized sex-specific biases, recommending adjusted BMI and NC thresholds for women. Studies by Xia et al. [32] and Chen et al. [33] also support lowering BMI thresholds (to 28 kg/m^2^) with NC adjustment (36 cm) in Asian women, which significantly improved sensitivity. The inclusion of BRI further strengthened this optimization by providing more precise assessment of sex-specific adiposity patterns. Prior research shows that while OSA prevalence is higher in men, postmenopausal women experience a marked increase in incidence [34,35], potentially related to hormonal effects on pharyngeal dilator muscle tone [36,37]. Additionally, severe female OSA patients are typically older than male counterparts. Our analysis found improved sensitivity in those aged ≤ 50 years (75.6% vs. 60.2%), suggesting better applicability in younger populations, possibly due to the greater contribution of visceral adiposity to OSA risk at earlier ages. Adjusting BMI thresholds in younger adults may thus prevent underdiagnosis of early-stage OSA [32].

This study has several limitations. First, it was conducted in a single tertiary sleep center in Southwest China, which may overrepresent symptomatic individuals with suspected OSA and higher cardiometabolic risk, limiting generalizability to community-based or primary-care populations in other regions of China. Second, craniofacial parameters such as mandibular retrognathia and tonsillar hypertrophy, which are important OSA predictors in non-obese individuals [38] and require imaging for accurate measurement, were not routinely collected and therefore not included, potentially underestimating model performance in certain subgroups. Third, the absence of long-term follow-up precludes evaluation of the tool’s predictive value for OSA-related outcomes such as cardiovascular events and diabetes. Fourth, unmeasured lifestyle factors (e.g., smoking, alcohol intake) and BRI measurement variability may have introduced residual confounding. Finally, menopausal status in women was not systematically verified beyond self-report, limiting detailed interpretation of age–sex interactions. These limitations highlight the need for multicenter, longitudinal studies that incorporate craniofacial and hormonal factors and validate the BRI-optimized STOP-Bang in broader Asian populations.

## 5. Conclusions

The BRI-optimized predicting model questionnaire showed better discrimination than the traditional tool in predicting severe OSA in Chinese adults, with particularly meaningful gains in women and younger adults. BRI appeared to perform better than BMI as an anthropometric predictor of severe OSA and may offer a practical and scalable enhancement to existing screening approaches in Asian populations. Further multicenter and prospective studies are needed to validate this model and determine its applicability across diverse clinical and community settings.

## Figures and Tables

**Table 1:** Baseline characteristics of all participants were stratified by AHI.

	All ^1^	AHI < 30 *n* = 3715 ^1^	AHI ≥ 30 *n* = 3864 ^1^	*p* Value ^2^
**Gender (%)**				**<0.001**
Female	2176.0 (28.7%)	1572.0 (42.3%)	604.0 (15.6%)	
Male	5403.0 (71.3%)	2143.0 (57.7%)	3260.0 (84.4%)	
**Snore (%)**				**<0.001**
Yes	6660.0 (87.9%)	2861.0 (77.0%)	3799.0 (98.3%)	
No	919.0 (12.1%)	854.0 (23.0%)	65.0 (1.7%)	
**Stop breathing (%)**				**<0.001**
Yes	3380.0 (44.6%)	1397.0 (37.6%)	1983.0 (51.3%)	
No	4199.0 (55.4%)	2318.0 (62.4%)	1881.0 (48.7%)	
**Leg movement (%)**				**0.026**
Yes	1586.0 (20.9%)	817.0 (22.0%)	769.0 (19.9%)	
No	5993.0 (79.1%)	2898.0 (78.0%)	3095.0 (80.1%)	
**Daytime sleepiness (%)**				**<0.001**
Yes	4019.0 (53.0%)	1763.0 (47.5%)	2256.0 (58.4%)	
No	3560.0 (47.0%)	1952.0 (52.5%)	1608.0 (41.6%)	
**Hypertension (%)**				**<0.001**
Yes	1658.0 (21.9%)	507.0 (13.6%)	1151.0 (29.8%)	
No	5921.0 (78.1%)	3208.0 (86.4%)	2713.0 (70.2%)	
**Arrhythmia (%)**				0.158
Yes	518.0 (6.8%)	238.0 (6.4%)	280.0 (7.2%)	
No	7061.0 (93.2%)	3477.0 (93.6%)	3584.0 (92.8%)	
**Coronary heart disease (%)**				0.120
Yes	422.0 (5.6%)	191.0 (5.1%)	231.0 (6.0%)	
No	7157.0 (94.4%)	3524.0 (94.9%)	3633.0 (94.0%)	
**Diabetes (%)**				**<0.001**
Yes	398.0 (5.3%)	137.0 (3.7%)	261.0 (6.8%)	
No	7181.0 (94.7%)	3578.0 (96.3%)	3603.0 (93.2%)	
**Cerebrovascular disease (%)**				**0.025**
Yes	316.0 (4.2%)	135.0 (3.6%)	181.0 (4.7%)	
No	7263.0 (95.8%)	3580.0 (96.4%)	3683.0 (95.3%)	
**Drinking (%)**				**<0.001**
Yes	3280.0 (43.3%)	1288.0 (34.7%)	1992.0 (51.6%)	
No	4299.0 (56.7%)	2427.0 (65.3%)	1872.0 (48.4%)	
**Smoking (%)**				**<0.001**
Yes	2440.0 (32.2%)	944.0 (25.4%)	1496.0 (38.7%)	
No	5139.0 (67.8%)	2771.0 (74.6%)	2368.0 (61.3%)	
**Age**				**<0.001**
Mean (SD)	43.9 (13.1)	41.9 (13.6)	45.7 (12.4)	
Median (Q1, Q3)	42.0 (34.0, 54.0)	40.0 (32.0, 52.0)	44.0 (36.0, 55.0)	
**Height**				**<0.001**
Mean (SD)	152.1 (48.1)	147.5 (52.4)	156.6 (43.2)	
Median (Q1, Q3)	167.0 (159.0, 172.0)	165.0 (157.0, 171.0)	168.0 (162.0, 173.0)	
**Weight**				**<0.001**
Mean (SD)	71.6 (14.4)	66.0 (12.8)	77.0 (13.8)	
Median (Q1, Q3)	70.0 (62.0, 80.0)	65.0 (56.0, 74.0)	75.0 (68.0, 85.0)	
**NC**				**<0.001**
Mean (SD)	38.1 (4.0)	36.4 (3.8)	39.7 (3.6)	
Median (Q1, Q3)	38.0 (35.0, 41.0)	36.0 (33.0, 39.0)	40.0 (38.0, 42.0)	
**BMI**				**<0.001**
Mean (SD)	25.5 (4.1)	23.9 (3.6)	27.1 (3.9)	
Median (Q1, Q3)	25.2 (22.9, 27.8)	23.7 (21.5, 25.8)	26.7 (24.6, 29.2)	
**BRI**				**<0.001**
Mean (SD)	4.4 (1.4)	3.9 (1.2)	4.9 (1.3)	
Median (Q1, Q3)	4.3 (3.5, 5.2)	3.8 (3.1, 4.5)	4.8 (4.1, 5.6)	
**WC**				**<0.001**
Mean (SD)	91.8 (11.6)	86.7 (10.8)	96.6 (10.2)	
Median (Q1, Q3)	92.0 (84.0, 99.0)	87.0 (79.0, 93.0)	96.0 (90.0, 102.0)	
**ESS**				**<0.001**
Mean (SD)	7.4 (5.6)	6.9 (5.6)	8.0 (5.6)	
Median (Q1, Q3)	6.0 (3.0, 11.0)	6.0 (2.0, 10.0)	7.0 (4.0, 12.0)	
**Stop-Bang**				**<0.001**
Mean (SD)	3.5 (1.6)	2.8 (1.5)	4.2 (1.4)	
Median (Q1, Q3)	3.0 (2.0, 5.0)	3.0 (2.0, 4.0)	4.0 (3.0, 5.0)	
**BRI-optimized STOP-Bang**				**<0.001**
Mean (SD)	4.0 (1.7)	3.2 (1.6)	4.8 (1.4)	
Median (Q1, Q3)	4.0 (3.0, 5.0)	3.0 (2.0, 4.0)	5.0 (4.0, 6.0)	

^1^ *n* (%); ^2^ Fisher’s exact test; Kruskal–Wallis rank sum test; Abbreviations: BMI, Body Mass Index; BRI, Body Roundness Index; NC, Neck circumference; WC, Waist Circumference; ESS, Epworth Sleepiness Scale; AHI, Apnea–Hypopnea Index.

**Table 2:** Baseline characteristics of all participants were stratified by BRI-Youden-cutoff.

	All ^1^	BRI < Youden *n* = 3020 ^1^	BRI ≥ Youden *n* = 4559 ^1^	*p* Value ^2^
**Gender (%)**				**<0.001**
Female	2176.0 (28.7%)	1212.0 (40.1%)	964.0 (21.1%)	
Male	5403.0 (71.3%)	1808.0 (59.9%)	3595.0 (78.9%)	
**Snore (%)**				**<0.001**
Yes	6660.0 (87.9%)	2368.0 (78.4%)	4292.0 (94.1%)	
No	919.0 (12.1%)	652.0 (21.6%)	267.0 (5.9%)	
**Stop breathing (%)**				**<0.001**
Yes	3380.0 (44.6%)	1136.0 (37.6%)	2244.0 (49.2%)	
No	4199.0 (55.4%)	1884.0 (62.4%)	2315.0 (50.8%)	
**Leg movement (%)**				**0.015**
Yes	1586.0 (20.9%)	590.0 (19.5%)	996.0 (21.8%)	
No	5993.0 (79.1%)	2430.0 (80.5%)	3563.0 (78.2%)	
**Daytime sleepiness (%)**				**<0.001**
Yes	4019.0 (53.0%)	1437.0 (47.6%)	2582.0 (56.6%)	
No	3560.0 (47.0%)	1583.0 (52.4%)	1977.0 (43.4%)	
**Hypertension (%)**				**<0.001**
Yes	1658.0 (21.9%)	304.0 (10.1%)	1354.0 (29.7%)	
No	5921.0 (78.1%)	2716.0 (89.9%)	3205.0 (70.3%)	
**Arrhythmia (%)**				**<0.001**
Yes	518.0 (6.8%)	140.0 (4.6%)	378.0 (8.3%)	
No	7061.0 (93.2%)	2880.0 (95.4%)	4181.0 (91.7%)	
**Coronary heart disease (%)**				**<0.001**
Yes	422.0 (5.6%)	83.0 (2.7%)	339.0 (7.4%)	
No	7157.0 (94.4%)	2937.0 (97.3%)	4220.0 (92.6%)	
**Diabetes (%)**				**<0.001**
Yes	398.0 (5.3%)	82.0 (2.7%)	316.0 (6.9%)	
No	7181.0 (94.7%)	2938.0 (97.3%)	4243.0 (93.1%)	
**Cerebrovascular disease (%)**				**<0.001**
Yes	316.0 (4.2%)	81.0 (2.7%)	235.0 (5.2%)	
No	7263.0 (95.8%)	2939.0 (97.3%)	4324.0 (94.8%)	
**Drinking (%)**				**<0.001**
Yes	3280.0 (43.3%)	1096.0 (36.3%)	2184.0 (47.9%)	
No	4299.0 (56.7%)	1924.0 (63.7%)	2375.0 (52.1%)	
**Smoking (%)**				**<0.001**
Yes	2440.0 (32.2%)	717.0 (23.7%)	1723.0 (37.8%)	
No	5139.0 (67.8%)	2303.0 (76.3%)	2836.0 (62.2%)	
**Age**				**<0.001**
Mean (SD)	43.9 (13.1)	40.4 (12.6)	46.1 (13.0)	
Median (Q1, Q3)	42.0 (34.0, 54.0)	39.0 (31.0, 50.0)	45.0 (36.0, 56.0)	
**AHI**				**<0.001**
Mean (SD)	36.9 (28.5)	22.9 (21.1)	46.2 (28.9)	
Median (Q1, Q3)	30.8 (11.9, 58.8)	15.8 (5.8, 34.1)	45.1 (20.8, 68.9)	
**Height**				0.888
Mean (SD)	152.1 (48.1)	150.6 (50.6)	153.1 (46.4)	
Median (Q1, Q3)	167.0 (159.0, 172.0)	167.0 (158.0, 173.0)	167.00 (160.0, 172.0)	
**Weight**				**<0.001**
Mean (SD)	71.6 (14.4)	63.4 (10.8)	77.0 (13.9)	
Median (Q1, Q3)	70.0 (62.0, 80.0)	63.0 (55.0, 70.0)	75.0 (68.0, 85.0)	
**NC**				**<0.001**
Mean (SD)	38.1 (4.0)	35.6 (3.4)	39.7 (3.6)	
Median (Q1, Q3)	38.0 (35.0, 41.0)	36.0 (33.0, 38.0)	40.0 (37.0, 42.0)	
**BMI**				**<0.001**
Mean (SD)	25.5 (4.1)	22.5 (2.6)	27.5 (3.7)	
Median (Q1, Q3)	25.2 (22.9, 27.8)	22.7 (20.9, 24.2)	27.06 (25.2, 29.4)	
**WC**				**<0.001**
Mean (SD)	91.8 (11.6)	81.7 (7.4)	98.4 (8.8)	
Median (Q1, Q3)	92.0 (84.0, 99.0)	83.0 (77.0, 87.5)	97.0 (93.0, 103.0)	
**ESS**				**<0.001**
Mean (SD)	7.4 (5.6)	6.6 (5.1)	8.0 (5.8)	
Median (Q1, Q3)	6.0 (3.0, 11.0)	6.0 (3.0, 10.0)	7.0 (3.0, 12.0)	
**Stop-Bang**				**<0.001**
Mean (SD)	3.5 (1.6)	2.6 (1.3)	4.1 (1.5)	
Median (Q1, Q3)	3.0 (2.0, 5.0)	3.0 (2.0, 3.0)	4.0 (3.0, 5.0)	
**BRI-optimized STOP-Bang**				**<0.001**
Mean (SD)	4.0 (1.7)	2.6 (1.3)	4.9 (1.4)	
Median (Q1, Q3)	4.0 (3.0, 5.0)	3.0 (2.0, 3.0)	5.0 (4.0, 6.0)	

^1^ *n* (%); ^2^ Fisher’s exact test; Kruskal–Wallis rank sum test; Abbreviations: AHI, Apnea–Hypopnea Index; BMI, Body Mass Index; BRI, Body Roundness Index; NC, Neck circumference; WC, Waist Circumference; ESS, Epworth Sleepiness Scale. Note: Continuous variables were expressed as mean ± SD or median (IQR) and compared using the Wilcoxon rank-sum test or Kruskal–Wallis test. Categorical variables were expressed as counts (%) and compared using Pearson’s chi-square test or Fisher’s exact test. A *p*-value < 0.05 was considered statistically significant.

**Table 3:** The comparison of predictive parameters in different data (AHI ≥ 30 events/h).

	AUC (95% CI)	Sensitivity (95% CI)	Specificity (95% CI)	PPV (95% CI)	NPV (95% CI)	AUC (95% CI), *p* (Delong)
**All data**						
STOP-Bang	0.747 (0.736–0.757)	66.6 (65.1–68.1)	71.0 (69.5–72.4)	70.5 (69.0–72.0)	67.2 (65.7–68.6)	-
BRI-optimized STOP-Bang	0.762 (0.751–0.772)	80.4 (79.1–81.6)	59.5 (57.9–61.0)	67.3 (66.0–68.7)	74.4 (72.8–76.0)	0.015 (0.011–0.019), ***p* < 0.001**
**Train data**						
STOP-Bang	0.735 (0.722–0.749)	66.3 (64.4–68.2)	69.4 (67.5–71.3)	70.2 (68.3–72.0)	65.5 (63.6–67.4)	-
BRI-optimized STOP-Bang	0.750 (0.737–0.763)	79.7 (78.1–81.3)	57.8 (55.7–59.8)	67.2 (65.5–68.9)	72.4 (70.3–74.5)	0.015 (0.010–0.019), ***p* < 0.001**
**Valid data**						
STOP-Bang	0.749 (0.729–0.770)	67.4 (64.6–70.2)	71.4 (68.5–74.2)	72.1 (69.2–74.9)	66.7 (63.7–69.5)	-
BRI-optimized STOP-Bang	0.766 (0.746–0.786)	80.9 (78.4–83.2)	59.0 (55.9–62.1)	68.4 (65.8–70.9)	73.8 (70.5–76.8)	0.017 (0.009–0.024), ***p* < 0.001**
**Test data**						
STOP-Bang	0.803 (0.772–0.835)	66.4 (60.5–72.0)	78.8 (74.5–82.6)	67.2 (61.2–72.8)	78.2 (74.0–82.1)	-
BRI-optimized STOP-Bang	0.820 (0.789–0.850)	84.1 (79.2–88.3)	69.9 (65.2–74.3)	64.6 (59.4–69.6)	87.1 (83.0–90.5)	0.017 (0.006–0.027), ***p* = 0.003**

**Table 4:** The comparison of predictive parameters in subgroup (AHI ≥ 30 events/h).

	AUC (95% CI)	Sensitivity (95% CI)	Specificity (95% CI)	PPV (95% CI)	NPV (95% CI)	AUC (95% CI), *p* (Delong)
**Male**						
STOP-Bang	0.684 (0.670–0.698)	71.5 (69.9–73.0)	56.8 (54.7–58.9)	71.6 (70.0–73.1)	56.7 (54.6–58.8)	-
BRI-optimized STOP-Bang	0.701 (0.687–0.715)	62.2 (60.5–63.8)	68.5 (66.4–70.4)	75.0 (73.3–76.6)	54.3 (52.4–56.2)	0.017 (0.011–0.022), ***p* < 0.001**
**Female**						
STOP-Bang	0.769 (0.749–0.790)	73.0 (69.3–76.5)	67.7 (65.3–70.0)	46.5 (43.3–49.7)	86.7 (84.7–88.6)	-
BRI-optimized STOP-Bang	0.792 (0.772–0.811)	84.3 (81.1–87.1)	59.6 (57.1–62.0)	44.5 (41.6–47.4)	90.8 (88.9–92.5)	0.023 (0.014–0.031), ***p* < 0.001**
**Age ≤ 50 years**						
STOP-Bang	0.768 (0.756–0.780)	60.2 (58.2–62.1)	79.5 (77.9–81.0)	73.1 (71.1–75.0)	68.3 (66.6–69.9)	-
BRI-optimized STOP-Bang	0.787 (0.775–0.799)	75.6 (73.9–77.3)	69.3 (67.5–71.0)	69.5 (67.7–71.2)	75.5 (73.7–77.1)	0.019 (0.014–0.024), ***p* < 0.001**
**Age > 50 years**						
STOP-Bang	0.693 (0.673–0.714)	53.5 (50.8–56.1)	74.7 (71.9–77.3)	73.9 (71.0–76.5)	54.6 (51.9–57.2)	-
BRI-optimized STOP-Bang	0.706 (0.685–0.726)	72.3 (69.9–74.7)	59.2 (56.2–62.2)	70.3 (67.9–72.7)	61.5 (58.4–64.6)	0.013 (0.006–0.020), ***p* < 0.001**

## Data Availability

The data that support the findings of this study are available from the corresponding author upon reasonable request due to privacy and ethical restrictions.
